# Backyard benefits? A cross-sectional study of yard size and greenness and children’s physical activity and outdoor play

**DOI:** 10.1186/s12889-021-11475-4

**Published:** 2021-07-15

**Authors:** Jessica Oakley, Rachel L. Peters, Melissa Wake, Anneke C. Grobler, Jessica A. Kerr, Kate Lycett, Raisa Cassim, Melissa Russell, Cong Sun, Mimi L. K. Tang, Jennifer J. Koplin, Suzanne Mavoa

**Affiliations:** 1 Murdoch Children’s Research Institute, Parkville, VIC Australia; 2 Department of Paediatrics, University of Melbourne, Parkville, VIC Australia; 3 School of Psychology, Faculty of Health, Deakin University, Burwood, VIC Australia; 4 Melbourne School of Population and Global Health, Faculty of Medicine, Dentistry, and Health Sciences, University of Melbourne, Parkville, VIC 3010 Australia

**Keywords:** Children, Physical activity, outdoor play, Yard, Greenness, Built environment

## Abstract

**Background:**

The home environment is the most important location in young children’s lives, yet few studies have examined the relationship between the outdoor home environment and child physical activity levels, and even fewer have used objectively measured exposures and outcomes. This study examined relationships between objectively assessed home yard size and greenness, and child physical activity and outdoor play.

**Methods:**

Data were drawn from the HealthNuts study, a longitudinal study of 5276 children in Melbourne, Australia. We used cross-sectional data from a sample at Wave 3 (2013–2016) when participants were aged 6 years (*n* = 1648). A sub-sample of 391 children had valid accelerometer data collected from Tri-axial GENEActive accelerometers worn on their non-dominant wrist for 8 consecutive days. Yard area and greenness were calculated using geographic information systems. Objective outcome measures were minutes/day in sedentary, light, and moderate-vigorous physical activity (weekday and weekend separately). Parent-reported outcome measures were minutes/day playing outdoors (weekend and weekday combined). Multi-level regression models (adjusted for child’s sex, mother’s age at the birth of child, neighbourhood socioeconomic index, maternal education, and maternal ethnicity) estimated effects of yard size and greenness on physical activity.

**Results:**

Data were available on outdoor play for 1648 children and usable accelerometer data for 391. Associations between yard size/greenness and components of physical activity were minimal. For example, during weekdays, yard size was not associated with daily minutes in sedentary behaviour (β: 2.4, 95% CI: − 6.2, 11.0), light physical activity (β: 1.4, 95% CI: − 5.7, 8.5) or MVPA (β: -2.4, 95% CI: − 6.5, 1.7), with similar patterns at weekends. There was no relationship between median annual yard greenness and physical activity or play.

**Conclusion:**

In our study of young children residing in higher socio-economic areas of Melbourne yard characteristics did not appear to have a major impact on children’s physical activity. Larger studies with greater variation in yard characteristics and identification of activity location are needed to better understand the importance of home outdoor spaces and guide sustainable city planning.

**Supplementary Information:**

The online version contains supplementary material available at 10.1186/s12889-021-11475-4.

## Background

Physical activity confers health and developmental benefits for young children, including mitigating the risks of obesity and non-communicable diseases [1, 2], and improved motor and cognitive development [3, 4]. Similarly, outdoor play, which encompasses both high and low levels of physical activity, also provides developmental benefits [5]. However, internationally, child physical activity levels are low [6, 7] and time playing outdoors is decreasing [8]. Therefore, identifying ways to promote physical activity and outdoor play in younger children is a public health priority.

The outdoor built environment is a promising mechanism for improving population physical activity levels. Substantial evidence indicates that the neighbourhood outdoor built environment can contribute to more active lifestyles in adults [9, 10]. It is also clear that young children are more active when outdoors [11, 12], and that living close to school is related to increased child physical activity levels via active travel [13, 14]. However, evidence supporting links between other aspects of the neighbourhood built environment and child physical activity levels is mixed [15, 16]. It may be that the residential neighbourhood is less relevant than the home outdoor environment, i.e. yard, as an important physical activity setting for young children before they go to school. Private yards provide immediate [17], safe and secure [18] access to outdoor spaces, especially when compared to public neighbourhood facilities [19, 20]. As such, yards could support increased outdoor time, which is linked to beneficial health behaviours and outcomes such as decreased sedentary behaviour, and increased physical activity and cardiorespiratory fitness [21].

Few studies have assessed relationships between yard size and physical activity levels or play, and the scant evidence is mixed. A cross-sectional study of 1596 2–5 year olds in metropolitan Perth, Australia, examined associations of parent-reported yard size with objectively measured moderate-to-vigorous physical activity (MVPA) and parent reported play [18]. While this study found little evidence of an association between yard size and MVPA, it found that for every 25m^2^ increase in yard size children spent an additional 1 min per day playing outdoors (β = 0.04, CI = 0.02–0.06) [18]. Conversely, a study of 280 children under 5 years of age and residing in metropolitan Adelaide, Australia, did not detect an association between yard size and outdoor play [22]. Similarly, a longitudinal study of 421 5–6 year-olds in metropolitan Melbourne, Australia, found little evidence of a relationship between yard size and parent-reported time outdoors [23].

Characteristics of yards may also play a role in child activity. A feature of yards that may be easy to modify is the level of vegetation. Yet there is a lack of studies assessing relationships between green and natural features of yards and physical activity or play. One exception is the Perth study, which showed that for every additional type of parent-reported natural feature/ play area present in the yard, children played outdoors for an additional 5 min per day (β = 5.19, CI = 2.96–7.43) [18]. To date, no studies have investigated objectively measured yard greenness and child physical activity or play.

To fill these evidence gaps, we assessed the relationship of objectively measured yard size and yard greenness with children’s (1) objectively measured physical activity and (2) parent reported outdoor play time, in a population-based sample of Australian 6-year-olds.

## Methods

### Study design and setting

The HealthNuts study is a population-based longitudinal study of allergic disease and general childhood health based in Melbourne, Australia. Detailed study methods have been described elsewhere [24–26]. Briefly, 5276 12-month-old-infants were recruited for the study when presenting for their 12-month council-run immunisations sessions (7134 participants were approached, 74% participation).

Our study used data from Wave Three of the HealthNuts study, which was collected between July 2013 and February 2016 when participants were 6 years of age. Children and their caregivers were invited to attend a HealthNuts study clinic at the Royal Children’s Hospital, Melbourne, for a comprehensive health assessment, and home visit assessments were offered to those who could not attend (61% participated, *n* = 3233/5276). Parents or guardians were also invited to complete the questionnaire, irrespective of whether they attended the health assessment (84% participated, *n* = 4455/5276). An additional questionnaire collected information on children’s outdoor activities (31% participated, *n* = 1648/5276).

From March 2015, children who attended the 6 year follow up clinical appointment were invited to participate in an accelerometer sub-study (number invited was not recorded). A total of 682 participants accepted the invitation to wear a tri-axial GENEActive (ActivInsights Ltd., Cambridgeshire, UK) accelerometer on their non-dominant wrist 24 h per day (including while sleeping, swimming, and bathing) for eight consecutive days [27]. Of these, 495 participants returned data, and 391 had valid accelerometer data (defined below). Parents recorded their children’s activities and accelerometer wear times.

Ethics approval was obtained from the Human Research Ethics Committee (HREC) of the Office for Children, Government of Victoria (CDF/07/492), Department of Human Services, Government of Victoria (10/07) and The Royal Children’s Hospital HREC (32294A). All methods were carried out in accordance with the relevant guidelines and regulations.

### Exposures

Participant addresses were geocoded (assigned a latitude and longitude) and yard size and median greenness at the residential address were calculated using ArcGIS Desktop 10.6.1 (ESRI, Redlands) as described in detail below.

#### Yard size

Yard size (m^2^) was calculated for each participant’s residential address by subtracting the area of the building footprint (area within roof outlines of all buildings) [28] from the area of the land parcel [29]. Therefore, the yard size includes all open space surrounding the house. We assume that if a child lived in an apartment building the yard size would be the common outdoor area for that building.

#### Yard greenness

Yard greenness was assessed using the Normalised Vegetation Index (NDVI), which ranges in value from − 1 to + 1. Higher values indicate higher levels of green vegetation and lower values generally indicate impervious surfaces, water, snow, cloud or moist soil [30]. We aimed to assess typical levels of vegetation in the yard over the year, rather than seasonal variation in greenness or the amount of green vegetation at the time of survey. Therefore, custom Google Earth Engine scripts were used to calculate median annual NDVI for each Landsat 8 satellite image (30 m by 30 m resolution) for each year between 2013 and 2017 [31]. Briefly, these scripts calculated the median NDVI for each cloud-free pixel across all images for the entire year. The annual median NDVI layers were downloaded and imported into GIS. Each participant was assigned an NDVI score based on the median NDVI 30 m × 30 m pixel that their address was located in. For each participant we used the annual median NDVI that corresponded to the year they participated in the Wave Three data collection.

### Outcomes

We evaluated both objective and parent report outcomes since no one measure captures all activity [32].

#### Physical activity

A detailed description of accelerometer data processing is provided elsewhere [27]. Briefly, accelerometer data were included if there was a minimum of 4 days wear, with at least one weekend/holiday day. Valid days had more than 10 h of wake wear time, or a minimum 16 wear hours. Phillips cut points were used to define sedentary < 488 g.min), light (1575 g.min), and moderate-to-vigorous physical activity (MVPA; > 4350 g.min) categories [33, 34]. Accelerometer data were cross-checked with the activity cards. Proportions of non wear sport time were assigned to sedentary (19%), light (46%) and MVPA (35%) categories based on a recent systematic review of youth physical activity time in structured settings [35]. If non-wear time was due to sleep, time was recategorized as sleep. Total physical activity was calculated by adding light physical activity and MVPA. Since the accelerometer was not linked to GPS we had no data on the location of different types of activity, including whether it was spent in their own outdoor area.

#### Outdoor play

Parents were asked: *“thinking about a typical week, about how many hours and minutes per day does this child spend outdoors for play / recreation outside of school hours?”* Parents reported separately for weekdays versus weekends. Six participants with implausible responses (> 7 h outdoors on weekdays, and > 12 h on weekends) were excluded.

### Confounders

Directed acyclic graphs were developed to identify the following possible confounders [36] (see Supplementary file 1 Figs. 1 and 2): parental ethnicity, parental education, parental age, socioeconomic status (SES), and parental attitudes to physical activity. Data were collected at Wave One or Wave Three questionnaires and were available for all confounders except for parental attitudes to physical activity. Due to missing paternal data we used maternal age, education, and ethnicity. Similarly, due to large numbers of missing household income data, we used neighbourhood SES as a proxy for individual SES. Mother’s ethnicity was categorised as Caucasian, Asian, Middle Eastern, or other. Mother’s highest level of education was categorised as year 12 or less, trade apprenticeship, technical diploma/certificate, university degree and postgraduate university degree, or other. Neighbourhood socioeconomic status was assessed using the socioeconomic indexes for areas (SEIFA) index of relative socioeconomic disadvantage (IRSD) score at the postcode level [26]. Additional covariates included child age and sex.

The presence of siblings [23, 37, 38] or pet dogs [39–41] were considered in the relationship between yard characteristics and physical activity and play, even though directionality was unknown (e.g., did parents choose a house with a large yard because they had a dog or did they get a dog because they had a large yard). Therefore, to assess whether these factors impacted on estimates, models were run with and without adjusting for sibling presence and dogs. Parents reported whether the participant had siblings living with them for more than half a week and whether the family had a dog.

### Statistical analysis

Prior to analysis the following transformations were applied: yard area was log-transformed due to skewness, and greenness was rescaled (multiplied by 10), yielding potential greenness scores of 0 to 10. While greenness scores can range from − 1 to 1, all participants in the study had positive greenness scores.

Participants were clustered in households (i.e., 146 sets of siblings from the same household at Wave Three) and geographic neighbourhoods. Therefore, associations between yard characteristics (size, greenness) and children’s physical activity and play were assessed using multi-level regression models. All models were specified with two levels: household level, and neighbourhood level. Neighbourhoods were defined as Statistical Area 3 (SA3), which have a population between 30,000–130,000 people, and are recognised as having a distinct social and economic identity [42].

Three versions of each multi-level regression model were run for each exposure and outcome combination. Model 1 was an unadjusted model specifying the exposure and outcome; clustered at two levels. Model 2 adjusted for a set of confounders established a priori: the child’s sex, the mother’s age at the birth of child, neighbourhood socioeconomic index (IRSD SEIFA), maternal education and maternal ethnicity. Model 3 additionally adjusted for the presence of siblings and pet dogs. Therefore, there were a total of 54 models based on: three versions, two exposures (yard size, greenness), and nine outcomes (weekday sedentary behaviour, weekday light physical activity, weekday moderate-vigorous physical activity, weekday total physical activity, weekend sedentary behaviour, weekend light physical activity, weekend moderate-vigorous physical activity, weekend total physical activity, parent reported minutes of outdoor play).

Since yard size was log-transformed, we divided the yard size coefficient by 10 and interpreted this as the effect of a 10% increase in yard size. The results from Model 2 are reported in the manuscript, with Models 1 and 3 presented in Additional file 1. We did not use any missing data techniques and analysed available data. Data analysis was performed using Stata version 15.1 (Statacorp, College Station, Texas, USA).

## Results

### Sample characteristics

HealthNuts participants who resided in Victoria and had valid residential address data that were able to be geocoded were eligible to be included in this study (*n* = 4672). The different data collection methods resulted in two analytic samples: (1) children with valid data from the outdoor activity questionnaire (*n* = 1648); and (2) children with valid accelerometer data (*n* = 391). Sociodemographic characteristics were consistent across the two subsamples, with both samples having slightly more boys than girls (approximately 51% males to 48% females), a high proportion of mothers of Caucasian descent (> 77%) and tertiary educated mothers (> 61%) (Table 1). Compared to the baseline Health Nuts wave 1 sample, our subsamples at wave 3 had similar numbers of boys and girls (wave 1: 50.5% male) but lived in more advantaged areas (wave 1: IRSD mean 1043.8).

**Table 1:** Socio-demographic and exposure characteristics of the analytic samples

	Sample 1 (*n* = 1648)	Sample 2 (*n* = 391)
Variable	Mean (SD) or %	Mean (SD) or %
Age of child (years)	6.3 (0.4)	6.2 (0.4)
Sex, %		
Male	50.3	50.6
Female	49.3	48.3
Missing	0.4	1.0
Mother’s ethnicity, %		
Caucasian	77.3	79.0
Asian	14.4	12.8
Middle Eastern	1.3	1.3
Other^1^	3.9	5.1^c^
Missing	3.0	1.8
Mother’s education, %		
Up to Year 12	11.8	13.3
Trade, Apprenticeship and other	20.3	23.3
Bachelor degree or higher	63.7	61.1
Other	0.7	0.0
Missing	3.4	2.3
Neighbourhood SES (IRSD score)^3^	1046.5 (50.3)	1050.8 (49.0)
Presence of Siblings, %		
Yes, and siblings live at home^2^	89.1	91.6
No siblings, or siblings not at home	8.5	7.4
Missing	2.4	1.0
Presence of dogs, %		
Have a dog	32.1	33.5
No dog	65.1	65.0
Missing	2.8	1.5
Yard Size		
Median (Inter Quartile Range), m^2^	374.4 (249.6–506.4)	357.4 (261.1–481.9)
% over 50m^2^	97.3	98.0
Greenness (rescaled NDVI), median (SD)^4^	3.9 (1.1)	4.0 (1.0)

Due to rounding percentages may not add to 100%

*IRSD* index of relative socioeconomic disadvantage, *NDVI* normalised difference vegetation index, *SD* standard deviation, *SES* socioeconomic status

^1^ Other includes those who noted their ethnicity as African, Aboriginal and Torres Strait Islander, as of a mixed ethnicity, or another ethnicity that was not listed as an option on the questionnaire

^2^ Living at home was defined as living at home for more than 50% of the week

^3^ IRSD score: national mean 1000 (SD: 100). A low IRSD score indicates relatively greater disadvantage and a high IRSD score indicates a relative lack of disadvantage

^4^ rescaled NDVI, a scale from 0 to 10 with 0 representing no vegetation and 10 representing full, healthy vegetation

**Fig. 1:**
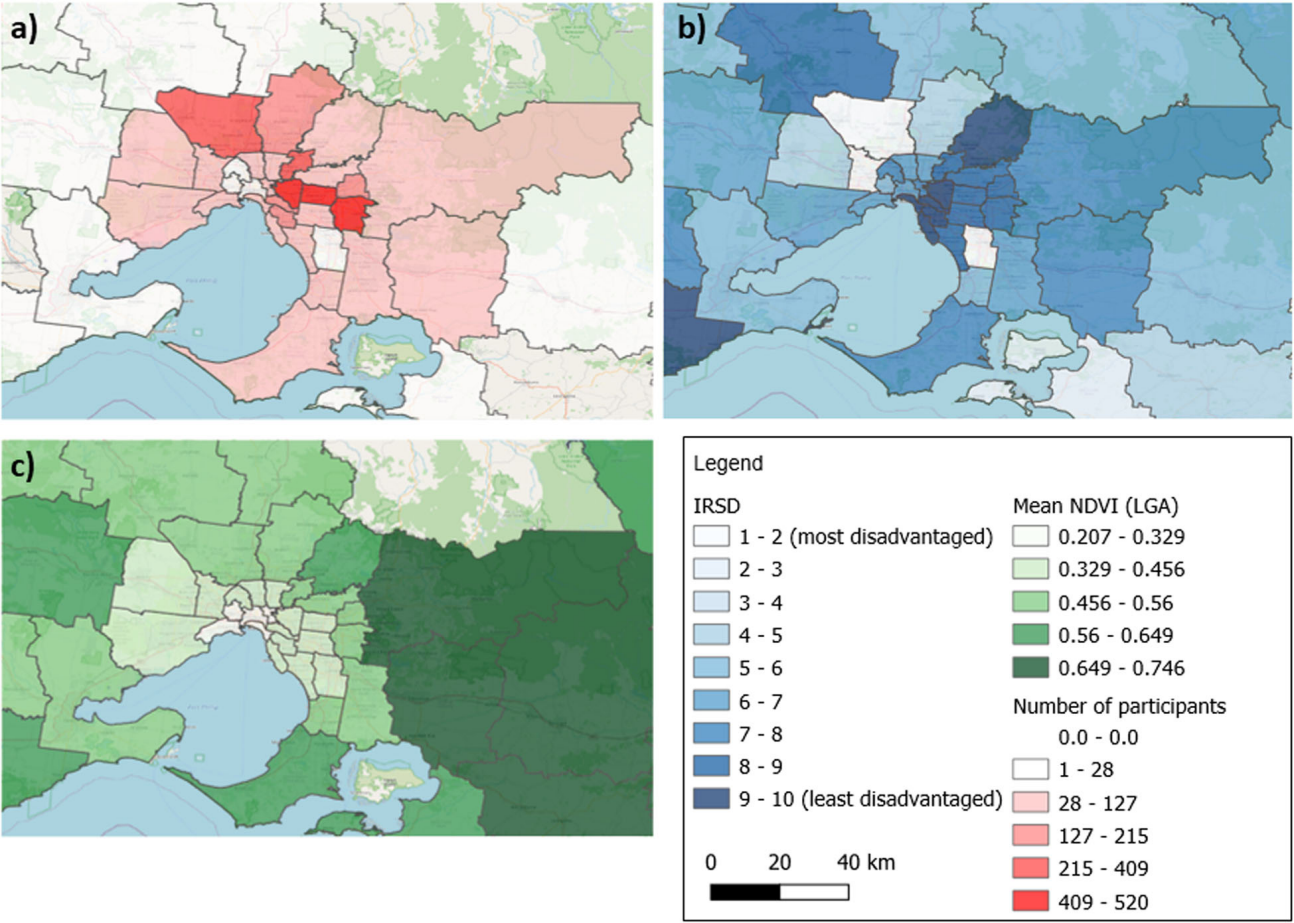
Location of **a**) participant residential addresses, **b**) areas of socioeconomic disadvantage and **c**) median annual NDVI (greenness)

Participants predominantly lived in neighbourhoods low in socioeconomic disadvantage, with a mean IRSD of 1047 to 1051 (Fig. 1), almost half a standard deviation above the national mean [43]. Median yard size was 374m^2^ in sample 1 and 357m^2^ in sample 2, which is relatively large and typical of older style Australian suburbs (in the order of 100-500 m^2^) as opposed to newer developments (20-200 m^2^) [44]. The rescaled median NDVI was 3.9 in sample 1 and 4.0 in sample 2 (Fig. 1).

### Physical activity and outdoor play

The accelerometer data showed that on average, children in this study spent 414.3 (SD: 69.7) mins/day in sedentary behaviour (6.9 h), 308.1 (SD: 55.3) mins/day in light physical activity (5.1 h), and 98.1 mins/day (SD: 34.8) in MVPA on weekdays (1.6 h; Table 2). Similar findings were seen for weekends (Table 2). On average parents reported their children played outside for 96.4 (SD: 59.5) mins/day.

**Table 2:** Results from multi-level regression models examining associations between yard characteristics and physical activity and outdoor play. Yard size β divided by 10 is minutes associated with a 10% increase in yard size. Greenness β is minutes associated with a 0.1 increase in NDVI

			Yard Size ^**a**^ (log(m^**2**^))		Greenness^**a,b**^ (rescaled to 0–10)	
	n	Mean (SD)	β (95% CI)	p	β (95% CI)	p
**Accelerometer measured physical activity**						
Accelerometer (mins/day) Weekdays						
Sedentary behaviour	371	414.3 (69.7)	-2.4 (−6.2, 11.0)	0.58	0.9 (− 6.0, 7.8)	0.95
Light physical activity	371	308.1 (55.3)	1.4 (−5.7, 8.5)	0.70	0.5 (−5.3, 6.3)	0.87
Moderate-to-vigorous physical activity	371	98.1 (34.8)	-2.4 (−6.5, 1.7)	0.25	−1.4 (−4.7, 1.9)	0.41
Total physical activity (LPA + MVPA)	371	406.8 (72.6)	−1.3 (− 10.5, 7.9)	0.79	−0.3 (−7.5, 6.8)	0.93
Weekends						
Sedentary behaviour	371	416.0 (89.4)	4.3 (−7.0, 15.6)	0.46	1.4 (−7.8, 710.5)	0.76
Light physical activity	371	303.0 (63.0)	1.2 (−6.6, 9.0)	0.78	3.5 (−2.8, 9.8)	0.27
Moderate-to-vigorous physical activity	371	94.1 (467)	0.1 (−5.7, 5.5)	0.97	−3.2 (−7.8, 1.3)	0.16
Total physical activity (LPA + MVPA)	371	397.3 (88.2)	1.2 (−9.8, 12.1)	0.83	1.1 (−7.4, 9.5)	0.80
**Parent reported outdoor play**						
Minutes spent outdoors playing (daily)	1535	96.4 (59.5)	**3.8 (1.0, 6.7)**	**0.009**	−1.6 (−4.5, 1.2)	0.26

*β* Regression coefficient, *CI* confidence interval, *SD* standard deviation, *p p*-value

^a^Adjusted for age, sex, socioeconomic position (SEIFA, maternal education), maternal ethnicity, and maternal age at birth

^b^Greenness calculated using NDVI; coefficient relates to a 10% increase in NDVI

### Associations of yard characteristics and physical activity

There was no evidence of associations between yard size and objectively measured total physical activity (Table 2), with almost all 95% confidence intervals crossing zero. For example, during weekdays, each 10% increase in yard size was associated with almost no increase in daily minutes in sedentary behaviour (β: 2.4, 95% CI: − 6.2, 11.0), light physical activity (β: 1.4, 95% CI: − 5.7, 8.5) or MVPA (β: -2.4, 95% CI: − 6.5, 1.7). On weekends there was a similar pattern for sedentary behaviour (β: 4.3, 95% CI: − 7.0, 15.6) and light physical activity (β: 1.2, 95% CI: − 6.6, 9.0).

Similarly, there was no evidence of associations between median annual greenness and physical activity (Table 2).

### Associations of yard characteristics and outdoor play

Since yard size is log-transformed, the β coefficient divided by 10 represents the number of minutes associated with a 10% increase in yard size. Therefore, for each 10% increase in yard size, parents reported 0.4 min more time outdoors playing (β: 3.8, 95% CI: 0.97, 6.74). Conversely, increased yard greenness was not associated with time in outdoor play (β: -1.5, 95% CI: − 3.7, 0.8).

Results from Model 3, which additionally adjusted for owning a dog and having siblings, were similar (see Supplementary Table 2).

## Discussion

### Principal findings

Our study examined relationships between yard characteristics and physical activity and play in young children. We detected evidence of an association between increased yard size and greater parent-reported outdoor play, but yard size was not associated with objectively measured physical activity. There was no association between median annual greenness and either physical activity or outdoor play.

### Interpretation considering previous studies

Participants in our study had, on average, relatively high levels of MVPA during weekdays (99.1 mins/day) compared to other studies of Australian children where only 21% meet physical activity guidelines of 60 min MVPA per day [45]. This may be partly due to our relatively high socio-economic sample with greater after school participation in sport [46]. Hence, any inferences based on this singular measure should be treated with caution.

Our results align with previous research that found that while larger yard sizes were not related to physical activity levels [18, 47], they were related to increased parental report of time in outdoor play [18, 22, 48]. These different results may be in part due to a lack of spatio-temporal specificity in the outcome measures. Our accelerometer based physical activity measures represented total physical activity regardless of location and so includes activity at home and away from home and both indoor and outdoor activity. While our outdoor play and outdoor time measures specified outdoor activity, they did not distinguish between outdoors at home and outdoors away from home. Our physical activity measures also lacked temporal specificity. For instance, our daily weekday physical activity measures included times when children are at school and unable to access their home yard. School is a substantial proportion of children’s activities during weekdays, and schools are common locations for children to accumulate MVPA [49]. Therefore, while our study was strengthened by specific objective exposure measures, the lack of spatio-temporal specificity in our outcome measures may have limited our ability to detect relationships between yard characteristics and physical activity and play [50, 51]. Future research would benefit from location specific survey questions and/or the use of GPS data (including linkage to accelerometer data) to assess activity within the yard, as well as distinguishing between school and non-school times during weekdays [52]. Despite these limitations, our data show that larger yard sizes were not a factor in overall physical activity.

Differing results for physical activity and outdoor play may also be partly due to differences in objective outcome measures and parent-reported measures. While parent-reported outdoor play is correlated with physical activity in pre-school aged children [53], parent-reported measures may also suffer known limitations of self-report measures [54]. For example, studies comparing parent report with objectively measured physical activity have shown that parent-reported time in different types of activities are consistently higher than objective measures [55, 56]. Our findings may also reflect a real distinction between physical activity, where MVPA is typically the focus, and outdoor play, which can incorporate a range of activity levels from sedentary to vigorously active. For instance, a systematic review of 2–5 year old’s physical activity and sedentary behaviour found that while only 15% of outdoor playtime in childcare was in MVPA, 53% was sedentary [57]. While low levels of sedentary behaviour and higher levels of MVPA have acknowledged health benefits [58, 59], it is also important to recognise that outdoor play - regardless of levels of physical activity - may also have developmental benefits [5].

Existing evidence has shown that greener, more natural environments facilitate more outdoor play in children since they provide more interesting play areas and promote enthusiastic, diverse and imaginative play [22, 60]. However, our study did not detect evidence of a relationship between yard greenness and parent-reported outdoor play. There are several possible explanations for this. First, we showed that our participants were predominantly located in the greenest suburbs in the city. Therefore, there may have been insufficient variation in greenness to detect any effects. Second, it is possible that our greenness measure was too coarse to capture the qualities of natural yards relevant to children’s activity. As such, future research aiming to quantitatively assess characteristics of yards could take advantage of higher resolution imagery and machine learning classification methods to address this issue (e.g., [61, 62]). However, it may also be that parent-reported measures of natural features of yards used in other studies are capturing more than just the physical environment (e.g., they may be capturing elements of parental values/perceptions of nature).

Finally, it is important to consider the role of yards as part of broader neighbourhood environmental and societal contexts. Smaller private outdoor spaces may be necessary as cities densify [63], with densification contributing to sustainability and population health benefits in cities, including increased adult physical activity [64, 65]. While a decrease in total available space is inevitable, it is important to note that reduced yard size can occur irrespective of the size of the lot. This maximisation of building area to lot size is increasingly common in Australia [66], and reversing this trend (e.g., by mandating smaller building footprints) is one way whereby private outdoor space could be protected in a densifying city. Additionally, many families are not able to afford to live in homes with large yards, as such another important consideration is the provision of shared private spaces in apartment buildings [67]. Our findings suggest that these factors may not necessarily come at the cost of children’s activity. Rather, urban design, shared indoor and outdoor spaces for activity, schools and public health solutions may be most beneficial in supporting young children’s physical activity and active play.

### Strengths and limitations

Our study has several strengths. We used objective measures of yard characteristics and physical activity, minimising the error and bias that can occur when participants report their environment [68]. We also examined both objective and parent-reported measures, providing a comprehensive assessment of children’s activity [69, 70]. There were several limitations. First, while the accelerometer sub-sample was larger than most other accelerometer studies with young children [71, 72], the sample was nonetheless small and the wide confidence intervals signify that the target parameters could be quite different from the point estimates. We also fitted 54 models in total, which meant that was substantial opportunity for spurious results. Second, the amount of outdoor play is a subjective measure and as discussed above may not equate to the construct of time spent being active. Third, we were not able to determine whether physical activity and outdoor play occurred within the yard. Fourth, this study only considered yard size and greenness – but not other characteristics of yards that may also have impact on children’s physical activity levels, for example, play equipment [18]. Finally, this cross-sectional study is not population representative and we were not able to determine the direction of any association. For instance, children who prefer to be outdoors may lead families to live in homes with larger outdoor spaces. Additionally, most participants lived in green, relatively affluent neighbourhoods.

## Conclusion

Little is known about the role of home outdoor environments in supporting child physical activity. Based on our sample of young children residing in higher socio-economic areas of Melbourne, yard characteristics did not appear to have a major impact on children’s physical activity. This suggests that child physical activity may not suffer as we densify our cities and reduce the size of yards. However, these findings need to be confirmed with larger studies that have greater variation in yard characteristics, are undertaken in different contexts, and can identify the location of activities.

## Supplementary Information

**Additional file 1.**

## Data Availability

The data that support the findings of this study are available on request from the HealthNuts study team (Murdoch Children’s Research Institute, Melbourne, Australia). The data are not publicly available since they contain information that could compromise research participant privacy/consent.

## Abbreviations

CI: confidence interval
GIS: geographic information systems
IRSD: index of relative socioeconomic disadvantage
LPA: light physical activity
MVPA: moderate-vigorous physical activity
NDVI: normalised difference vegetation index
OR: odds ratio
SA3: Statistical Area 3
SD: standard deviation
SEIFA: socioeconomic indexes for areas
SES: socioeconomic status
