# Supplementary material for: Backyard benefits? A cross-sectional study of yard size and greenness and children’s physical activity and outdoor play
Source: BMC Public Health. 2021 Jul 15;21:1402. doi: 10.1186/s12889-021-11475-4 (PMC8283889; doi:10.1186/s12889-021-11475-4)
Supplement: Supplementary file 1 — Additional file 1. [file 12889_2021_11475_MOESM1_ESM.docx]

**Backyard Benefits? A cross-sectional study of yard size and greenness and children’s physical activity and outdoor play**

Jessica Oakley^1,2^, Rachel L. Peters^1^, Melissa Wake^1,2^, Anneke C. Grobler^1,2^, Jessica A. Kerr^1,2^, Kate Lycett^1,2,3^, Raisa Cassim^1,4^, Melissa Russell^4^, Cong Sun^1^, Mimi L.K. Tang^1,2^, Jennifer J. Koplin^1^, Suzanne Mavoa^1,4^*

**Affiliations**

^1^Murdoch Children’s Research Institute, Parkville, VIC Australia
^2^Department of Paediatrics, University of Melbourne, Parkville, VIC Australia
^3^School of Psychology, Faculty of Health, Deakin University, Burwood, VIC Australia
^4^Melbourne School of Population and Global Health, University of Melbourne, Parkville, VIC Australia


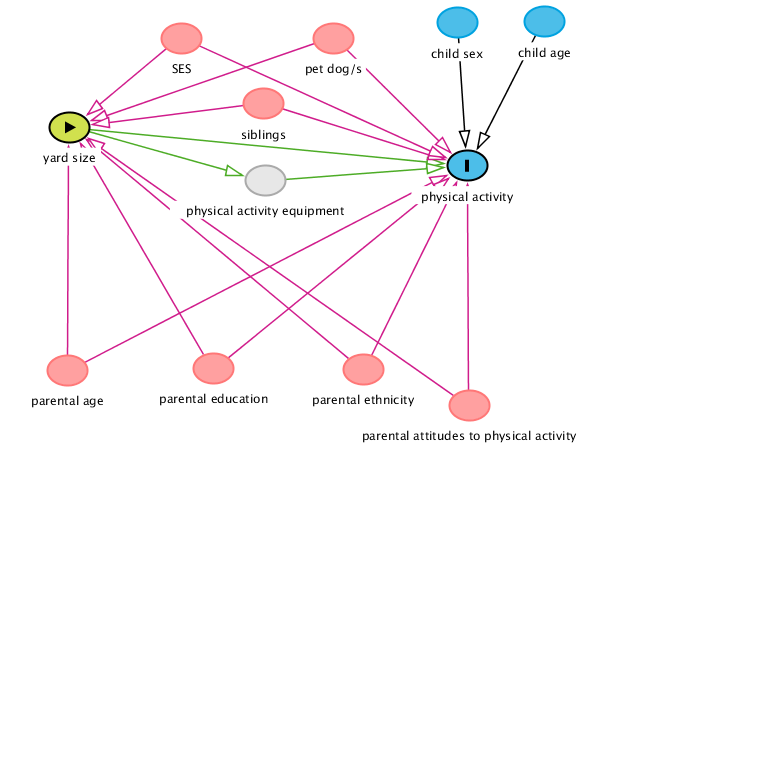


Supplementary Figure 1. Directed acyclic graph of the relationship between yard size and child physical activity and play.


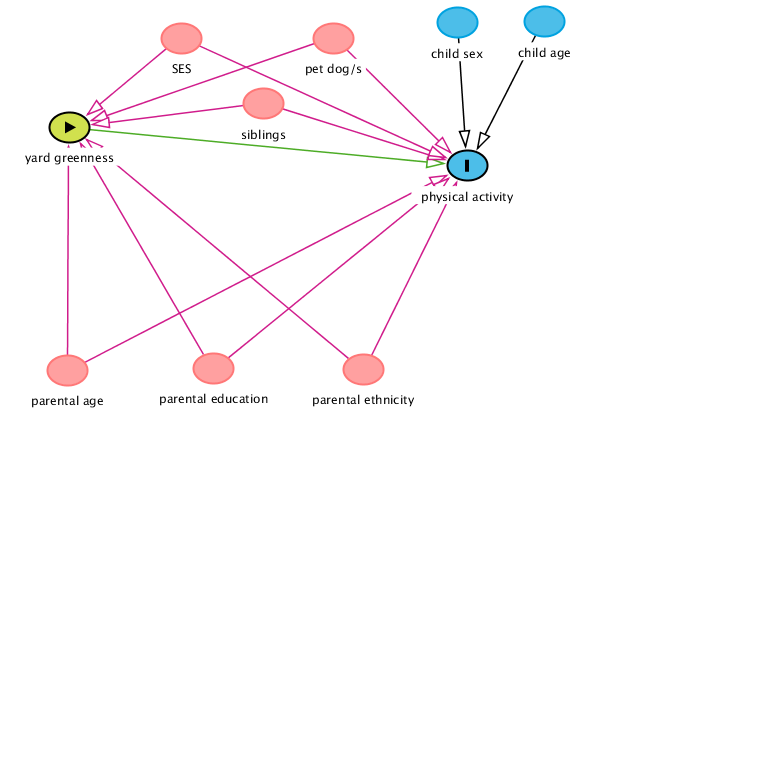


Supplementary Figure 2. Directed acyclic graph of the relationship between yard greenness and child physical activity and play.

Supplementary Table 1. Model 1: Regression coefficients from unadjusted multi-level regression models estimating relationships between yard characteristics and physical activity and play. Yard size β divided by 10 is minutes associated with a 10% increase in yard size. Greenness β is minutes associated with a 0.1 increase in NDVI.

|  |  |  |  |  | **Yard Size ^a^ (log(m^2^))** | |  | **Greenness^b^ (rescaled to 0-10)** | |
| --- | --- | --- | --- | --- | --- | --- | --- | --- | --- |
|  |  | **n** | **Mean (SD)** |  | **β (95% CI)** | **p** |  | **β (95% CI)** | **p** |
| **Accelerometer measured physical activity** |  |  |  |  |  |  |  |  |  |
| Accelerometer (mins/day) |  |  |  |  |  |  |  |  |  |
| Weekday |  |  |  |  |  |  |  |  |  |
| Sedentary behaviour |  | 382 | 414.3 (69.7) |  | 0.1 (-8.1, 7.8) | 0.97 |  | -1.5 (-8.2, 5.3) | 0.67 |
| Light physical activity |  | 382 | 308.1 (55.3) |  | 0.7 (-5.7, 7.1) | 0.83 |  | 0.4 (-5.9, 5.15) | 0.88 |
| Moderate-to-vigorous physical activity |  | 382 | 98.5 (34.8) |  | -0.1 (-4.2, 3.9) | 0.95 |  | 0.3 (-3.2, 3.8) | 0.88 |
| Total physical activity (LPA + MVPA) |  | 382 | 406.8 (72.6) |  | 0.4 (-8.0, 8.8) | 0.92 |  | 0.6 (-6.5, 7.6) | 0.88 |
| Weekends |  |  |  |  |  |  |  |  |  |
| Sedentary behaviour |  | 382 | 416.0 (89.4) |  | 3.0 (-7,4, 13.4) | 0.57 |  | -0.2 (-9.1, 8.7) | 0.86 |
| Light physical activity |  | 382 | 303.0 (63.0) |  | -0.5 (-7.8, 6.9) | 0.90 |  | 0.9 (-5.3, 7.2) | 0.77 |
| Moderate-to-vigorous physical activity |  | 382 | 94.1 (46.7) |  | 1.2 (-4.3, 6.7) | 0.67 |  | -1.9 (-6.6, 2.8) | 0.42 |
| Total physical activity (LPA + MVPA) |  | 382 | 397.3 (88.2) |  | 0.6 (-9.6, 10.8) | 0.91 |  | 0.1 (-8.4, 8.6) | 0.98 |
| **Parent reported outdoor play** |  |  |  |  |  |  |  |  |  |
| Minutes spent outdoors playing (daily) |  | 1610 | 96.4 (59.5) |  | **4.9 (2.0, 7.8)** | **0.001** |  | -1.6 (-4.4, 1.3) | 0.29 |
| ^b^Greenness calculated using NDVI, NDVI transformed into deciles; coefficient relates to a 0.1 change in NDVI.  **β** : Regression coefficient; CI: confidence interval; SD: standard deviation; OR: Odds Ratio; p: p-value. | | | | | | | | | |

Supplementary Table 2. Model 2: Regression coefficients from multi-level regression models estimating relationships between yard characteristics and physical activity and play.

|  |  |  |  |  | **Yard Size^a^ (log(m^2^))** | |  | **Greenness^a,b^ (rescaled to 0-10)** | |
| --- | --- | --- | --- | --- | --- | --- | --- | --- | --- |
|  |  | **n** | **Mean (SD)** |  | **β (95% CI)** | **p** |  | **β (95% CI)** | **p** |
| **Accelerometer measured physical activity** |  |  |  |  |  |  |  |  |  |
| Accelerometer (mins/day) |  |  |  |  |  |  |  |  |  |
| Weekday |  |  |  |  |  |  |  |  |  |
| Sedentary behaviour |  | 369 | 414.3 (69.7) |  | 1.8 (-6.9, 10.4) | 0.69 |  | -0.9 (-6.2, 7.8) | 0.81 |
| Light physical activity |  | 369 | 308.1 (54.3) |  | 1.8 (-5.3, 9.0) | 0.61 |  | 0.7 (-5.1, 6.4) | 0.82 |
| Moderate-to-vigorous physical activity |  | 369 | 98.1 (34.8) |  | -2.0 (-6.1, 2.0) | 0.33 |  | -1.5 (-4.8, 1.8) | 0.37 |
| Total physical activity (LPA + MVPA) |  | 369 | 406.8 (72.6) |  | -1.2 (-10.4, 8.0) | 0.79 |  | -0.3 (-7.4, 6.8) | 0.93 |
| Weekends |  |  |  |  |  |  |  |  |  |
| Sedentary behaviour |  | 369 | 416.0 (89.4) |  | 3.6 (-7.7, 15.0) | 0.53 |  | -0.5 (-8.6, 9.7) | 0.91 |
| Light physical activity |  | 369 | 303.0 (63.0) |  | 1.5 (-6.3, 9.4) | 0.70 |  | 4.0 (-2.3, 10.3) | 0.21 |
| Moderate-to-vigorous physical activity |  | 369 | 94.1 (46.7) |  | 0.2 (-5.4, 5.9) | 0.93 |  | -3.0 (-7.6, 1.5) | 0.19 |
| Total physical activity (LPA + MVPA) |  | 369 | 397.3 (88.2) |  | 1.9 (-9.1, 12.8) | 0.74 |  | 1.7 (-6.7, 10.1) | 0.69 |
| **Parent reported outdoor play** |  |  |  |  |  |  |  |  |  |
| Minutes spent outdoors playing (daily) |  | 1527 | 96.41 (59.51) |  | **3.57 (0.69, 6.45)** | **0.015** |  | -1.9 (-4.8, 1.0) | 0.20 |
| ^a^Adjusted for age, sex, socioeconomic status (SEIFA, maternal education), maternal ethnicity, maternal age at birth, presence of siblings and presence of pet dogs;  ^b^Greenness calculated using NDVI, NDVI transformed into deciles; coefficient relates to a 0.1 change in NDVI.  **β** : Regression coefficient; CI: confidence interval; SD: standard deviation; OR: Odds Ratio; p: p-value. | | | | | | | | | |
